# Role of the Gut–Liver Axis in Driving Parenteral Nutrition-Associated Injury

**DOI:** 10.3390/children5100136

**Published:** 2018-09-25

**Authors:** Christine Denton, Amber Price, Julie Friend, Chandrashekhara Manithody, Keith Blomenkamp, Matthew Westrich, Vindhya Kakarla, William Phillips, Joseph Krebs, Armando Salim Munoz Abraham, Hector Osei, Ajay Kumar Jain

**Affiliations:** 1 Department of Pediatrics, Saint Louis University School of Medicine, 1465 South Grand Blvd., St. Louis, MO 63104, USA; christine.denton@health.slu.edu (C.D.); amber.price@health.slu.edu (A.P.); julie.friend@health.slu.edu (J.F.); chandrashekhara.manithody@health.slu.edu (C.M.); keith.blomenkamp@health.slu.edu (K.B.); matthew.westrich@slu.edu (M.W.); vindhya.kakarla@slu.edu (V.K.); phillipsw@slu.edu (W.P.); joey.krebs@slu.edu (J.K.); 2 Department of Surgery, Saint Louis University School of Medicine, 1 N. Grand Blvd., St. Louis, MO 63103, USA; salim.munozabraham@health.slu.edu (A.S.M.A.); hector.osei@health.slu.edu (H.O.)

**Keywords:** parenteral nutrition, total parenteral nutrition, fibroblast growth factor 19, farnesoid x receptor, glucagon like peptide, chenodeoxycholic acid, cholesterol 7 alpha-hydroxylase 1

## Abstract

For decades, parenteral nutrition (PN) has been a successful method for intravenous delivery of nutrition and remains an essential therapy for individuals with intolerance of enteral feedings or impaired gut function. Although the benefits of PN are evident, its use does not come without a significant risk of complications. For instance, parenteral nutrition-associated liver disease (PNALD)—a well-described cholestatic liver injury—and atrophic changes in the gut have both been described in patients receiving PN. Although several mechanisms for these changes have been postulated, data have revealed that the introduction of enteral nutrition may mitigate this injury. This observation has led to the hypothesis that gut-derived signals, originating in response to the presence of luminal contents, may contribute to a decrease in damage to the liver and gut. This review seeks to present the current knowledge regarding the modulation of what is known as the “gut–liver axis” and the gut-derived signals which play a role in PN-associated injury.

## 1. Introduction

Parenteral nutrition (PN) is the method of nutritional delivery that bypasses the gastrointestinal tract and intravenously administers key elements of nutrition, such as amino acids, glucose, vitamins, minerals, and lipids. When all nutritional needs are met in this way, the term total parenteral nutrition (TPN) is used [1,2,3]. For individuals unable to tolerate enteral feeding, such as those with impaired gut function, this therapy can be crucial in preventing or correcting malnutrition [1]. Thus, TPN has remained an essential modality of nutrition delivery in neonatal, pediatric, and adult populations worldwide since its clinical establishment in the mid-20th century [4,5,6].

Although some individuals receive adjunctive or short-term delivery of parenteral nutrition during hospitalization, perioperative management, or home care [3,4], others may require long-term administration of PN as a life-sustaining therapy, such as those with intestinal failure [7]. In these individuals, regardless of the underlying etiology of disease, the gut is unable to maintain proper homeostasis of fluids, electrolytes, and nutrients. One well-recognized subset of patients with intestinal failure includes those with short bowel syndrome (SBS), which is a consequence of extensive bowel resection secondary to events such as trauma, congenital defects, ischemic injury, inflammatory bowel disease, or necrotizing enterocolitis [8,9]. Patients with SBS often present with intestinal failure, in which digestive and absorptive processes are compromised, therefore requiring long-term use of PN to ensure nutrition and survival [7,10].

Despite its evident value, PN has also been associated with serious, and sometimes fatal, complications. Parenteral nutrition-associated liver disease (PNALD), also referred to as intestinal failure-associated liver disease (IFALD), has been well-described as a cholestatic liver injury with a wide spectrum of presentations, including hepatic inflammation, steatosis, dyslipidemia, glucose intolerance, and hepatic fibrosis [10,11,12]. In some patients, progression to liver failure or death may occur. Additionally, many studies using animal models to evaluate the effect of TPN on the gut have observed intestinal mucosal atrophy [13,14,15].

**Figure 1:**
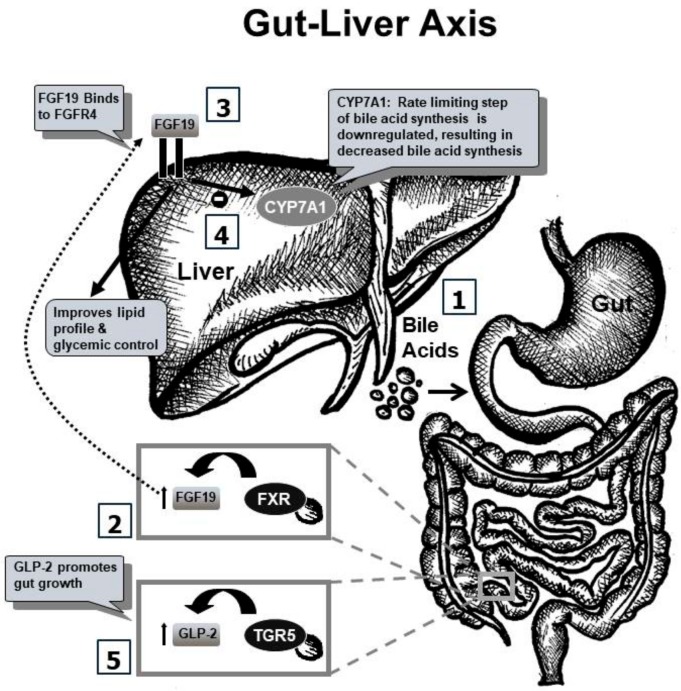
Gut–Liver Axis. 1. Bile acids are secreted into the small intestine and travel through the gut. 2. FXR, with its highest concentration in the terminal ileum, is activated by bile acids. This results in FGF19 secretion into portal circulation. 3. FGF19 binds with its receptor, FGFR4 in hepatocytes. 4. FGF19 regulates CYP7A1, the rate-limiting step of bile acid synthesis, resulting in a decrease in bile acid synthesis, thus, hepatic bile acids are modulated by gut signaling. 5. TGR5 is activated by bile acids, leading to an increase in GLP-2, which promotes gut growth. CYP7A1: Cholesterol 7 alpha-hydroxylase; GLP-2: Glucagon-like peptide 2; FGF19: Fibroblast growth factor 19; FGFR4: Fibroblast growth factor receptor 4; FXR: Farnesoid X receptor; TGR5: Takeda G-protein-coupled receptor 5.

Research regarding mechanistic pathways and ameliorative modalities in parenteral nutrition-associated injury remains a major focus in the field of gastroenterology and hepatology. Although several theories have been proposed, the pathophysiology and etiology of PN-associated injury remains unknown. Recent animal and human data reveal that an alteration in gut-derived signals may occur with administration of TPN in the absence of enteral nutrition, leading to gut and liver injury. This hypothesis is driven by the observation that hepatic injury is decreased if even a small percentage of nutrition can be provided enterally [8,16]. This challenges previous theories suggesting that components of PN cause direct injury to the liver and gut. Based on the concept that gut-derived signals stimulated by intraluminal nutrients can maintain liver health, it is hypothesized that in the state of TPN and absence of enteral nutrition, there is disruption in the enterohepatic axis, leading to injury [17]. The purpose of this review is to present the relevant basic science and clinical research focusing on the gut–liver crosstalk in the context of TPN-associated injury (Figure 1). Although some studies have shown that lipid emulsions have toxic properties [18,19] and prematurity [20,21,22] may also play a role in PN-associated injury in neonatal patients, these theories are outside the purview of our discussion.

## 2. Evidence Supporting the Importance of Enteral Nutrition and Luminal Content

### 2.1. Hepatic Injury

The severity of TPN liver injury is typically reduced upon introduction of enteral nutrition. This was demonstrated in a study published by Javid et al., who concluded that the level of conjugated bilirubin, which is classically elevated in PNALD, normalized after initiation of full enteral nutrition in a cohort of 12 infants on PN for a duration of 5 ± 1 months. These findings supported the aggressive weaning of PN and establishment of enteral autonomy [16].

A retrospective analysis of 172 neonates, aimed to ascertain risk factors contributing to PN-associated injury, reported significant differences in the development of cholestasis, depending on the day enteral feedings were started. They concluded that early initiation of enteral nutrition was protective against such injury [2]. In a clinical study evaluating liver disease in extremely low birth weight infants, differences in cholestasis were noted between conservative (n = 77) and aggressive (n = 85) enteral nutritional regimens. Although the aggressive regimen introduced amino acids and lipids into parenteral nutrition earlier compared to the conservative regimen, it also included initiation of enteral nutrition immediately after birth with larger volume advancements. This was compared to the conservative regimen, which initiated half-strength feeds when neonates were “clinically stable” with smaller feed advancements. The aggressive regimen resulted in a significant decrease in cholestasis, as well as a marked improvement in postnatal growth [23].

Animal models evaluating hepatocellular damage provide evidence linking the duration of TPN and the severity of liver injury. In fact, a significant increase in cholestasis, glucose intolerance, and dyslipidemia were demonstrated even after a short duration of TPN use when compared to animals receiving enteral nutrition [20,24,25,26].

### 2.2. Gut Injury

Compelling evidence has shown that in normal enteral feeding, key regulatory signals are released in response to the presence of intraluminal nutrients. Recent research postulates that TPN-associated gut injury may result from the lack of luminal nutrient delivery, thus leading to an alteration in the release of regulatory signals derived from a lack of luminal receptor activation [13,14]. One important observation seen with TPN use is atrophy of gut mucosa. Although it was initially described with chronic TPN use, recent evidence indicates that atrophic changes may occur as early as 24–48 h after TPN initiation.

Using a porcine model, Niinikoshi demonstrated that, compared to enteral nutrition animals, TPN piglets had a rapid reduction of intestinal blood flow within 8 h of TPN initiation. After 24 h, TPN piglets had reduced jejunal inducible nitric oxide synthase (iNOS) activity, protein abundance, small intestinal weight, and villous height. Cell proliferation and DNA mass were decreased, while apoptosis increased after 48 h on TPN [14]. In a study by Buchman et al., eight healthy human volunteers were placed on parenteral support for 14 days followed by 5 days of enteral nutrition. Jejunal biopsies via endoscopy were taken before and after TPN, as well as after 5 days of enteral feeding. The study noted a decrease in total mucosal thickness after TPN and also noted a decrease in villus height [27].

## 3. Gut–Liver Axis

Given the data supporting the importance of enteral nutrition in reducing TPN-associated injury, and the potential role of the gut–liver axis, it is essential to determine potential mechanistic pathways driving this effect.

### 3.1. Portal System

The gut–liver axis theory is supported by the anatomical connection between the intestine and liver. The portal vein, which provides greater than two-thirds of the blood flow to the liver [28], is essential for hepatocellular survival. This direct link enables movement of gut-derived nutrients, signaling molecules, metabolic end products, and toxins into the portal system. Without this blood flow, hepatocellular function fails [29].

Some studies have reported a relationship between portal flow and enteral nutrition. In a study by Omata et al., reduced blood flow to the small intestine and portal vein was observed during TPN administration, resulting in a reduction in hepatic mononuclear cells (MNCs), potentially contributing to hepatocellular dysfunction. Upon initiation of enteral feeds, parenteral-induced reduction of portal vein and small intestine blood flow, as well as hepatic MNCs, were restored to the levels of the control group [29].

### 3.2. Gut Microbiota

#### 3.2.1. Human Gut *Microbiota*

Recent human and animal studies have provided evidence demonstrating the role of the gut microbiota in modulation of disease pathology [30]. With over 100 trillion commensal organisms and thousands of species, the human intestinal microbiota genome, or the gut microbiome, represents 200,000–300,000 genes. This is 10 times that of the aggregate human host genome [31].

When viewed collectively, the gut microbiota is a “super-organism” that can perform critical physiologic functions that benefit the host, such as enhancement of the mucosal immune system, production of short-chain fatty acids from undigested carbohydrates, production of vitamins, and metabolism of bile salts. These functions can subsequently affect health [32,33].

#### 3.2.2. TPN and Modulation of Gut Microbiota

It is hypothesized that a change in the composition of the gut microbiota occurs when enteral nutrition is absent during TPN, resulting in altered luminal metabolism and luminal signaling [34].

As the dominant phyla in the gut, *Firmicutes* and *Bacteroidetes* have been studied in relation to host health, and an alteration in their abundance and/or ratio [30] has been evaluated in different disease states, such as obesity [35] and nonalcoholic fatty liver disease [36]. Investigation of the changes in gut microbial composition has carried over into research focused on parenteral nutrition. For instance, in a study by Hodin et al., an observed shift in small intestinal luminal microbiota occurred in rats receiving TPN for 14 days. TPN animals were shown to have a significant decrease in the ratio of *Firmicutes* to the total bacteria as compared to enterally fed animals, as well as a decrease in the ratio of *Firmicutes* to *Bacteroidetes.* However, neither a significant decrease in the abundance of *Bacteroidetes* nor a change in the total abundance of bacteria was seen in TPN animals [37]. Conversely, mice receiving PN in a study by Heneghan et al. exhibited a reduction in *Firmicutes* and an increase in *Bacteroidetes* compared to chow controls. They postulated that a paucity of enteral nutrition correlated with these changes in microbial composition during PN [38].

Although an anatomically and functionally heterogenous population, individuals with short bowel syndrome (SBS), have been shown to have intestinal dysbiosis. One study revealed that variability in the etiology of SBS could result in differential makeups of the gut microbiota. Adult individuals with jejunocolic anastomosis had an increase in *Proteobacteria,* while those with jejunoileal anastomosis had an increase in *Lactobacillus* [39]. A study by Joly et al. observed a relative proliferation of *Proteobacteria*, as well as a decrease in *Bacteroidetes* [40]. The gut microbiota of pediatric SBS patients has also been evaluated. For example, Engelstad et al. studied the intestinal microbiota in pediatric short bowel syndrome (SBS) patients compared to intestinal length. They noted that those with the shortest remaining bowel required more parenteral nutrition support, had larger relative abundance of *Proteobacteria*, and had decreased gut microbial diversity [41].

Alterations in the normal gut microbiota have been studied in relation to inflammatory responses in hosts. For instance, *Proteobacteria* has been associated with a proinflammatory state. In the above study by Engelstad et al., two patients with very little remnant bowel who required >95% of their calories via PN had elevated levels of interleukin-8 (IL-8). They were also found to have gut microbiota compositions with >75% *Proteobacteria.* [41] In addition, *Bacteroides fragilis*, a species within the phylum *Bacteroidetes,* has been associated with intestinal inflammation and increased intestinal permeability [42,43,44,45]. It is hypothesized that such gut alterations enable bacterial translocation across the intestinal mucosa, resulting in cytokine- and endotoxin-mediated modulation of key hepatobiliary receptors [46,47,48]. Significantly higher levels of interleukin-6 (IL-6) and tumor necrosis factor (TNF) are also observed [49,50,51].

In addition, antibiotic therapy may alter a normal gut microbiota, cause dysbiosis, or have the potential to induce the proliferation of pathogenic organisms, such as *Clostridium difficile* [52]. Some studies have demonstrated that antimicrobials, such as metronidazole and tetracycline, may reduce livery injury during parenteral nutrition. When metronidazole and tetracycline are administered per os during TPN use, a significant reduction in inflammation-induced hepatic injury is observed [53,54,55].

Although there are not sufficient data demonstrating its role in this population, fecal microbiota transplantation has been shown to be an effective treatment in recurrent *C. difficile* infections through the administration of gut microbiota from healthy donors. Given the observed changes in the gut microbiota during parenteral nutrition, future therapies may focus on restoring or altering the intestinal microbial community, with the goal of decreasing parenteral nutrition-associated injury [56].

### 3.3. Hepatobiliary Receptors and Transporters

Several hepatobiliary signaling pathways have been evaluated to further explore the gut–liver crosstalk and its subsequent disruption during TPN infusion.

#### 3.3.1. FXR-FGF19 Axis

Farnesoid X receptor (FXR), also known as NR1H4, is a bile acid receptor present throughout the gut, with its highest concentration in the terminal ileum. During enteral nutrition, bile acids in the gut activate FXR, resulting in enhanced secretion of the metabolic hormone fibroblast growth factor-15 (FGF-15) and its human orthologue fibroblast growth factor-19 (FGF19) into portal circulation [20]. In addition to regulating the metabolism of glucose and lipids in the body, FGF19 plays a significant role in the modulation of cholesterol 7 alpha-hydroxylase 1 (CYP7A1). An elevation of circulating FGF-19 leads to suppression of the CYP7A1 gene that encodes the rate-limiting step in bile acid synthesis. This inhibition is also suppressed by the activation of the short heterodimer partner (SHP)-liver receptor homologue 1 (LRH1) cascade [57,58]. When bile acids bind to FXR, transcription of SHP occurs and inactivates LRH, inhibiting CYP7A1 via negative feedback [59,60].

Loss of normal enterohepatic circulation of bile acids in TPN therapy contributes to mucosal gut atrophy and PNALD through the decrease in FGF19 concentration and modulation of bile acid synthesis. Several studies have demonstrated an association between decreased FGF19 levels in TPN [20,61]. For example, Mutanen et al. conducted a prospective study of 52 patients with intestinal failure (median age = 6 years) and assessed serum FGF19 levels after 10 months of TPN. The authors noted significantly lower FGF19 concentrations in intestinal failure patients receiving TPN when compared to FGF19 levels in healthy matched controls. Interestingly, FGF19 concentrations were further decreased in patients with ileal resection, with a positive correlation of serum FGF19 concentrations to remaining ileum (*p* = 0.028). Lower FGF19 levels in patients with portal inflammation or fibrosis (*p* = 0.013) were also seen, implying that FGF19 alterations may contribute to liver injury [62].

In a study by Lundasen et al., FXR agonist chenodeoxycholic acid (CDCA), or bile acid sequestrant resin cholestyramine, was given to adult human subjects and serum fasting levels of FGF19 were measured. CYP7A1 enzyme activity level was also measured using a surrogate marker, 7alpha-hydroxy-4-cholesten-3-one (C4). The enteral CDCA treatment group had increased FGF19 levels by 250% from baseline and a 26% decrease in C4 levels. In contrast, the cholestyramine treatment group had an 18-fold elevation in C4 level, accompanied by an 87% reduction in FGF19 levels from baseline [63].

Due to the presence of FXR in several organs, tissue-specific knockout animal models have been used to study the importance of gut FXR. In a mouse model by Kim et al., liver FXR knockout (FxrL) and gut FXR knockout (FxrIE) mice were treated with a selective FXR agonist, GW4064. Significant suppression of CYP7A1 was noted in FxrL mice, but not in the FxrIE mice. This demonstrates that CYP7A1 modulation was mediated specifically via gut FXR and helps further support the theory of gut–liver crosstalk [64]. In fact, mechanistic effects are further highlighted with gut resection as in SBS, which impairs the gut–liver crosstalk, precluding hepatic improvement despite gut FXR agonists, underscoring the importance of the gut–liver axis [8].

#### 3.3.2. TGR5–GLP Axis

Multiple studies have demonstrated that an absence of enteral nutrition can result in intestinal mucosal atrophy and increased intestinal permeability. Rodent studies, such as one performed by Ekelund et al., showed that exogenously given bile acids result in gut mucosal proliferation [15,25]. A large animal (porcine) study revealed that treatment with a bile acid receptor agonist preserves gut mucosa [20].

The gut’s response to treatment with bile acids has been linked to a metabolic protein, glucagon-like peptide-2 (GLP-2), which has emerged as an important gut-trophic factor [25]. In a human study, adult patients with SBS on TPN were treated with a subcutaneous GLP-2 analogue that resulted in a reduction of both the volume and the number of days of parenteral nutrition support [65]. In another study comprising 102 patients, comparing 55 patients with intestinal failure (median age 4.2 years) to 47 healthy control patients, increasing levels of serum glucagon-like peptide (GLP) correlated with a significantly reduced requirement of parenteral nutrition, thus supporting GLP’s role in regulating intestinal adaptation [66].

TGR5, a G-coupled protein receptor, is localized in gut mucosal crypts and, when activated by bile acids, mediates the secretion of GLP-2 by enteroendocrine cells [57,67,68,69]. Recent data suggest that the glucagon like peptide-1 (GLP-1), which is also under TGR5 modulation, affects insulin regulation, glucose homeostasis, and hepatic steatosis [70]. GLP-1 is the primary gut hormone responsible for enhanced insulin responses to nutrient ingestion and maintenance of glucose homeostasis [71]. In fact, glucose intolerance as well as impaired insulin sensitivity has been noted in animal TPN studies [20]. This TGR5–GLP axis further strengthens the gut–liver crosstalk theory.

### 3.4. GLP-2 and EGF

Epidermal growth factor (EGF) is known to play an important role in intestinal health. A murine study evaluated rats receiving parenteral nutrition in combination with GLP-2, EGF, or GLP-2 + EGF. Their results revealed increased small intestinal cellular proliferation, as well as increases in small intestinal weight by 75%, 43%, and 116%, respectively. They concluded that the administration of both GLP-2 and EGF together had a synergistic effect on intestinal growth [72]. A study by Feng et al. showed that exogenous administration of either GLP-2 or EGF resulted in protective effects against gut mucosal atrophy in TPN-treated mice. They also evaluated the interdependency of EGF and GLP-2 in reducing atrophic changes in the gut. To test this, they performed a reciprocal inhibitor study. In TPN animals receiving exogenous EGF, the GLP-2 antagonist, GLP-2 (3–33), was given. In TPN animals receiving exogenous GLP-2, the EGFR kinase inhibitor was given. In both groups, the protective effects of the exogenously given EGF or GLP-2 was blocked by the reciprocal inhibitor. As a result, villus height, crypt depth, and crypt cell proliferation remained at TPN levels. They concluded that there is likely interconnectivity between signaling pathways of EGF and GLP-reducing gut mucosal atrophy [73]. In fact, luminal signaling is known to affect both EGF and GLP-2, which further highlight the systemic roles of gut-derived signaling [74].

## 4. Expert Commentary: Five-Year View and Conclusion

Although parenteral nutrition remains lifesaving, enthusiasm for such therapy is tempered due to its unfortunate association with significant morbidity. Although several mechanisms for PN-related injury have been proposed, there is growing research supporting the theory that impaired gut signaling due to an absence of luminal nutrients is a major driver for both hepatic and gut injury. This is exciting, as targeting the impaired enterohepatic crosstalk could greatly improve outcomes for a large segment of the population partially or fully dependent on PN for survival.

Key hepatobiliary receptors, including farnesoid X receptor (FXR) as well as TGR5, appear to play a critical role in the pathogenesis of TPN injury. Current studies have shown that decreased synthesis of the hepatoprotective FGF19, as well as decreased synthesis of the gut growth hormone, GLP-2, occurs with TPN infusion, likely from a lack of enteral nutrition.

FXR signaling is known to regulate the secretion of FGF19, which modulates CYP7A1, the rate-limiting step of bile acid synthesis. This process is also disrupted with TPN. Such defects in bile acid metabolism cause hepatocellular injury, leading to cellular apoptosis, and ultimately result in fibrotic changes in the liver. Unfortunately, progression to end-stage liver disease may occur if these patients are left untreated. Without a liver transplantation, and in some cases combined liver and small bowel transplantation, such patients cannot survive.

GLP-2 is known to modulate gut growth and its integrity. The deficiency of GLP-2 levels in TPN therapy results in gut atrophy. This can lead to increased bacterial translocation, which may ultimately result in bacteremia and endotoxin-mediated liver injury, shock, or death. Thus, there has been strong support for the addition of trophic feeds in patients receiving PN, with the goal of decreasing bacterial translocation and liver injury.

In addition, results from TPN animal studies utilizing exogenous stimulation of FXR and TGR5 show improvement in both histology and serum injury markers. It is very likely that the same mechanisms contribute to TPN-associated injury in humans, and therapeutic targeting of these receptors by potent agonists could help improve the safety of parenteral nutrition.

Several FXR and TGR5 agonists are currently under development. FXR agonists have been used in clinical studies evaluating liver histology in disease processes other than TPN injury, providing data for their safety and feasibility of administration. Additionally, several GLP analogues are under development, some of which are currently being used in adult patients with short bowel syndrome. Over the next 5 years, focused research into the impairment of gut–liver and gut–systemic signaling pathways may foster development of preventative and ameliorative strategies for PN-associated injury.

## 5. Key Issues

1. Parenteral nutrition (PN) has been a successful method for the intravenous delivery of nutrients and remains an essential therapy for individuals with intolerance of enteral feedings or impaired gut function. PN provides nourishment via the intravenous administration of nutritional components, such as amino acids, glucose, vitamins, minerals, and lipids. When nutritional needs are solely met via such intravenous therapy, the resulting process is called total parenteral nutrition (TPN).
2. TPN remains an essential modality of nutrition delivery worldwide and is commonly used in neonates and pediatric and adult patients with lost or impaired gut function. TPN use has grown exponentially over the last few decades.
3. Despite the several benefits of TPN therapy, its use is associated with adverse effects. Parenteral nutrition-associated liver disease (PNALD) is a cholestatic liver disease, which can also present with hepatic inflammation, steatosis, dyslipidemia, glucose intolerance, and/or fibrosis. Significant gut mucosal atrophy has also been shown to occur in association with TPN infusion in several animal studies.
4. The pathophysiology and etiology of TPN-associated injury remains largely unknown despite several theories.
5. Research into ameliorative therapies and mechanistic pathways continues to be a major focus in the field of gastroenterology and hepatology.
6. Recent studies reveal that an alteration in gut-derived signals may occur with administration of TPN in the absence of enteral nutrition. This hypothesis is driven by the observation that hepatic injury is decreased if even a small percentage of nutrition can be provided enterally.
7. Based on the concept that gut-derived signals, stimulated by intraluminal nutrients, can maintain liver health, it is hypothesized that in the state of TPN, the enterohepatic axis is disrupted, thus leading to injury.
8. Current data, including work from our lab, support the theory that altered gut–liver crosstalk with TPN and bile acids have emerged as key regulators of injury. In fact, treatment with bile acid receptor agonists seems to ameliorate TPN-associated injury in animal models.
9. Translating research from bench to clinical targeting of the gut-derived signaling appears to be key in developing novel approaches in mitigating TPN-associated injury and restoring the effectiveness of this lifesaving therapy.
